# Editorial: Emerging mutations in colorectal cancer development and progression

**DOI:** 10.3389/fonc.2024.1469129

**Published:** 2024-08-28

**Authors:** Paul Ruff

**Affiliations:** School of Clinical Medicine, University of the Witwatersrand Faculty of Health Sciences, Johannesburg, South Africa

**Keywords:** colorectal cancer, mutations, MSI-H, MMR-D, EGFR-1, Ras, BRAF, signaling pathways

Colorectal cancer (CRC) is one of the most common malignancies and causes of cancer-related deaths, being the third most common cancer and the second most common cause of cancer death worldwide. GLOBOCAN estimated that in 2022 CRC constituted 9.6% of the approximately 2.5 million cancers diagnosed worldwide, as well as 9.3% of the approximately 1.8 million cancer deaths worldwide (1). CRC is primarily sporadic in nature although about 3-4% of patients have germline mutations as part of the Lynch syndrome (previously known as hereditary non-polyposis coli) or familial polyposis coli (APC). Sporadic CRC may be in part related to lifestyle risk factors, especially diets high in fat and low in fiber, as well as lack of exercise, obesity, alcohol consumption, and tobacco smoking. It is estimated that by the year 2035, the total number of deaths from colon and rectal cancer will increase by 71.5% and 60%, respectively. Overall survival (OS) at 5 years after primary diagnosis in stage I–II patients is about 87-90%, decreasing to about 68–72% in stage III patients, and further dropping to about 11–14% in stage IV metastatic CRC (mCRC). Although CRC has primarily been a disease seen in high-income countries (HICs), it is rapidly increasing in low-middle-income countries (LMICs) possibly related to dietary changes to so-called Western diets, high in fat and low in fiber, and yet unknown gene mutations. Indeed, CRC also seems to be being diagnosed at younger ages in LMICs with mean ages of about 10 years younger than in HICs, especially in patients of African descent (2, 3).

CRC is a clinical and etiologically heterogeneous disease that is characterized by clinical, diagnostic, and therapeutic differences based on tumor-sidedness probably related to its embryologic origin, from either the midgut or the hindgut, as well as various germline and somatic molecular mutations in global gene expression. Studies have found that the cumulative impact of genetic and epigenetic mutations in tumor suppressor genes, oncogenes, and DNA mismatch-repair (MMR) genes may be a potential precursor to the onset of the disease, the response to treatment, as well as its progression and outcome (4–6).

Germline mutations and deletions in the APC gene, and DNA MMR genes including MLH1, MSH2, MSH6, and PMS2, which result in increased microsatellite instability (MSI-H) have been highlighted to play a key role in the etiology of CRC and result in a predisposition to the disease in some families, particularly those with the Lynch syndrome. Somatic mutations of DNA MMR genes also result in increased (MSI-H) in about 12% of CRC tumors. The remaining 85% of CRCs are however microsatellite stable (MSS) as their DNA repair genes are proficient. Genetic alterations have been found to influence the dysregulation of various signaling pathways which lead to drug resistance, inhibition of apoptosis, and progression of tumor invasion and proliferation leading to colorectal tumor growth and metastasis, as well as resistance to both chemotherapeutic and targeted treatments.

Somatic mutations of EGFR-1 signal transduction pathway genes, especially of the RAS family kinases, especially KRAS and NRAS, as well as BRAF kinases, as well as of MEK and ERK/MAPK, appear to be important in tumorigenesis and cancer progression. In addition, alterations in the PI3K/PTEN/AKT/mTOR pathway, especially of PI3K and PTEN, also appear to play an important role in CRC development and progression, associated with resistance to treatment.

Today therapeutic algorithms for CRC contain endoscopic and surgical resection, systemic adjuvant chemotherapy, radiation therapy, palliative chemotherapy, targeted therapy, including monoclonal antibodies and signal-transduction kinase inhibitors, and immunotherapy. Due to the poor outcomes of numerous colorectal patients to existing therapeutic approaches and since CRC survival is highly dependent on primary diagnosis, staging, and early treatment, known significant biomarkers that can predict beneficial responses as early as possible are critical. In addition to biomarkers guiding early diagnosis and treatment, monitoring of these biomarkers during the course of the disease is essential in evaluating the response to treatment and helping treatment decision-making.

In patients with metastatic CRC (mCRC), 5-fluorouracil (5FU)/irinotecan/oxaliplatin-based chemotherapy (FOLFIRI or FOLFOX) in combination with either EGFR-1 or VEGF targeted therapy has improved median overall survival (OS) from less than 1 year before the year 2000 to 2 years or more today.

The addition of cetuximab, a chimeric IgG1 anti-EGFR-1 monoclonal antibody, to 5FU/irinotecan-based chemotherapy (FOLFIRI) in newly diagnosed mCRC patients increased the median progression-free survival from 8.4 months to 9.9 months (HR 0.70; 95% CI 0.56-0.87; p=0.0012) and the median overall survival from 20 months to 23.5 months (HR 0.80; 95% CI 0.67-0.95; p=0.0094) in KRAS exon 2 wild-type patients (~45% of patients) but showed no benefit in the KRAS mutant patients (4).

The addition of panitumumab, a fully human IgG2 anti-EGFR-1 monoclonal antibody to 5FU/oxaliplatin-based chemotherapy (FOLFOX) in newly diagnosed mCRC increased the median progression-free survival (PFS) from 7.9 months to 10.1 months (HR 0.72; 95% CI 0.58-0.90; p=0.004) and the median overall survival (OS) from 20.2 months to 26 months (HR 0.78; 95% CI 0.62-0.99; p=0.04) in RAS wild-type patients, with 17% of the KRAS exon 2 wild-type patients having other RAS mutations. The addition of panitumumab to FOLFOX chemotherapy in RAS mutant patients however showed a negative benefit in both PFS and OS (5).

The addition of pembrolizumab, a humanized anti-PD1 monoclonal antibody to chemotherapy in mCRC showed (6).

Until recently it has been very difficult to follow the development and implications of such mutations as well as their relationship to disease outcomes, due to the difficulties and high risks of repeated biopsies to obtain new tumor tissue. The advent of “liquid biopsies” with the ability to analyze nanogram amounts of circulating cell-free tumor DNA (cfDNA/ctDNA) has made it now possible to follow patients’ disease course and outcomes and correlate them with molecular changes (7).

Recent studies have shown a correlation between CRC disease outcomes and the emergence of new mutations of EGFR-1 signaling pathway genes with a relationship between higher mutant allele frequency and poorer patient outcomes. In addition, emergent mutations of the extracellular ligand-binding domain may negatively affect the binding of anti-EGFR-1 monoclonal antibodies to specific epitopes in this domain (8, 9).

This Research Topic aims to generate a discussion regarding the emergence of mutations of various components of the EGFR-1 and other signaling pathways in the field of colorectal cancer, how it influences the disease, and the impact it has on patient outcomes.
